# The chemical chaperone sodium 4-phenylbutyrate improves the secretion of the protein C_A267T_ mutant in CHO-K1 cells trough the GRASP55 pathway

**DOI:** 10.1186/s13578-015-0048-4

**Published:** 2015-10-09

**Authors:** Maria Eugenia Chollet, Ellen Skarpen, Nina Iversen, Per Morten Sandset, Grethe Skretting

**Affiliations:** Department of Hematology, Oslo University Hospital Rikshospitalet, 0424 Oslo, Norway; Research Institute of Internal Medicine, Oslo University Hospital Rikshospitalet, Box 4950 Nydalen, 0424 Oslo, Norway; Core Facility for Advanced Light Microscopy, Institute for Cancer Research, Oslo University Hospital, Montebello, 0379 Oslo, Norway; Department of Medical Genetics, Oslo University Hospital Ulleval, Oslo, Norway; Institute of Clinical Medicine, University of Oslo, Oslo, Norway

**Keywords:** Protein C, Misfolding, Chemical chaperones, GRASP55, Mutant

## Abstract

Some inherited coagulation factor deficiencies are caused by intracellular retention or degradation of misfolded proteins, and chemical chaperones have been shown to reverse protein misfolding. The purpose of the present study was to investigate whether chemical chaperones may improve secretion of the protein C_A267T_ (PC_A267T_) mutant in a cellular model. Using stably transfected Chinese hamster ovary cells (CHO-K1) expressing PC_A267T_ we demonstrate that sodium 4-phenylbutyrate (PBA) increased the secretion of PC_A267T_ by approximately 4-fold in comparison with untreated cells, and that this secretion seemed to follow an unconventional pathway via the Golgi reassembly stacking protein (GRASP55).

Dear Editor,

In order to determine whether chemical chaperones were able to restore secretion of the PC_A267T_ mutant, we treated Chinese hamster ovary cells (CHO-K1) stably expressing PC wild type (PC_wt_) or PC_A267T_ [1] with 3 different compounds: Sodium 4-phenylbutyrate (PBA), trimethylamine *N*-oxide (TMAO) and taurourosdeoxycholic acid (TUDCA). While no effect was seen with TMAO or TUDCA, treatment with PBA for 48 h enhanced the secretion of PC_A267T_. Our data indicate that PBA can rescue the trafficking of this mutant PC via an unconventional pathway involving the Golgi reassembly stacking protein GRASP55.

Several diseases are caused by defective protein folding leading to excessive protein degradation by proteasomes or to aggregation of misfolded proteins inside or outside the cell [2].

In some inherited coagulation factor deficiencies, failure of the mutant protein to adopt its properly folded state has been demonstrated. Our group has previously described the protein C_A267T_ (PC_A267T_) mutant in a patient with congenital PC deficiency and recurrent deep vein thrombosis [3]. We found that this mutant, PC_A267T_, was retained in the endoplasmic reticulum (ER), most likely due to misfolding of the protein. This caused ER stress, activation of the unfolded protein response and apoptosis [1, 4]. Moreover, in hemophilia A, a domain-specific misfolding in the FVIII A3 domain caused ER retention of the mutant FVIII with poor secretion of the functional protein [5].

Recently, several therapeutic strategies using small molecules able to correct or prevent misfolding of a protein, have been explored. Amongst them are chemical chaperones, which are low molecular mass compounds that effectively are able to inhibit the formation of misfolded structures and reverse the intracellular retention of misfolded proteins [6]. Therefore, chemical chaperones could be effective in reversing misfolding of blood coagulation proteins.

Since our previous studies have shown a defective trafficking of the PC _A267T_ mutant [4] we tested a possible effect of chemical chaperones on the secretion of this mutant protein. Thus, CHO-K1 cells stably expressing PCwt and the mutant PC_A267T_ were treated with PBA at 1, 2, and 5 mM for 48 h, trimethylamine *N*-oxide (TMAO) at 25 and 50 mM for 24 h and taurourosdeoxycholic acid (TUDCA) at 0.2 and 0.5 mM for 16 h. Untreated cells were used as control. The concentrations of PC antigen in the culture medium were measured using the Zymutest Protein C kit and the total protein concentration of the cell lysates was measured by the BCA Protein Assay kit. PC antigen levels in culture medium were normalized to the total concentration of protein of the respective cell lysates. Three independent experiments were performed in triplicates. Results were tested for statistical significance using one-way ANOVA or Student’s t test. P values <0.05 were considered statistically significant. GraphPad Prism version 5 was used for statistical analysis.

No effect was observed with TMAO or TUDCA treatment. In contrast, treatment with PBA for 48 h enhanced the secretion of PC_A267T_ in a dose dependent manner (Fig. 1). A small increase in the secretion of PC_wt_ was also observed (data not shown).

**Fig. 1:**
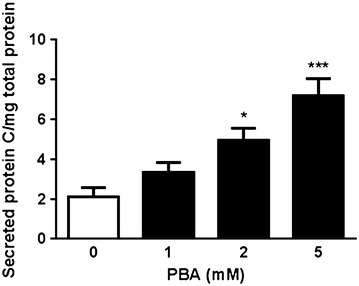
PBA improved the secretion of PC_A267T_. CHO-K1 cells stably expressing PC_A267T_ were incubated with indicated concentrations of PBA for 48 h. The levels of PC in the cell medium were determined using an ELISA kit. The results are presented as % PC relative to the PC levels in a plasma pool from healthy subjects (set as 100 %) and adjusted to the total protein concentrations in corresponding cell lysates. Results from three independent experiments are presented as mean ± SEM relative to untreated control cells. *p < 0.05, ***p < 0.0005

Studies have shown that PBA can reverse the cellular mislocalization or misfolding of proteins. For example, PBA treatment increased the secretion of ER retained α1-ATZ in a cell and mouse model of mutant alpha 1-antitrypsin-Z (α1-ATZ) [7]. Thus, for the PC_A267T_ mutant, the positive effect of PBA on secretion of the mutant protein can probably be related to a stabilization of the protein conformation by PBA, which can facilitate the trafficking of the protein. This might be a general effect of PBA since the secretion of the PC_wt_ was also found to be slightly increased.

Since our group previously demonstrated that PC_A267T_ was retained in the ER [4], we hypothesized that PBA treatment could restore the normal intracellular trafficking of the mutated protein. Thus, we assessed the intracellular localization of PC_A267T_ after PBA treatment by confocal microscopy using double staining for PC (rabbit polyclonal anti-PC) and a marker for ER (mouse monoclonal anti-protein disulfide isomerase, PDI), or for PC and a Golgi marker (mouse monoclonal anti-GM-130). Alexa Fluor 488 goat anti-rabbit and Alexa fluor 568 donkey anti-mouse were used as secondary antibodies. Prior to PBA treatment we found that PC_A267T_ was predominantly localized in the ER compartment (Fig. 2A, a–c) as previously described [4], while another fraction of PC_A267T_ was found associated with Golgi (Fig. 2B, a–c). After treating the cells with PBA, PC_A267T_ showed a more cytoplasmic distribution pattern (Fig. 2A, B, compare a and d). To assess whether the cytoplasmic localization of PC_A267T_ could be caused by its redistribution from the ER and/or Golgi compartments, we did co-localization analysis on merged images of PC_A267T_ combined with either PDI or GM130 before and after PBA treatment. As shown in Fig. 2C, the fraction of PC_A267T_ that co-localized with PDI was strongly reduced (Manders from 0.76 to 0.47) after treatment with PBA. In addition, the fraction of PC_A267T_ that co-localized with the Golgi compartment was also reduced (Manders from 0.4 to 0.16). No differences were observed in the fraction of PC_wt_ that co-localized with the ER after PBA treatment (data not shown). For the fraction of PC_wt_ that co-localized with Golgi, only a slight and non-significant reduction was observed (Manders from 0.29 to 0.19) after PBA treatment. Thus, our results suggest that PBA caused a redistribution of PC_A267T_ from both ER and Golgi to the cytoplasm. The small effect of PBA on the redistribution of PC_wt_ might be due to a small fraction of the normal protein that is incorrectly folded.

**Fig. 2:**
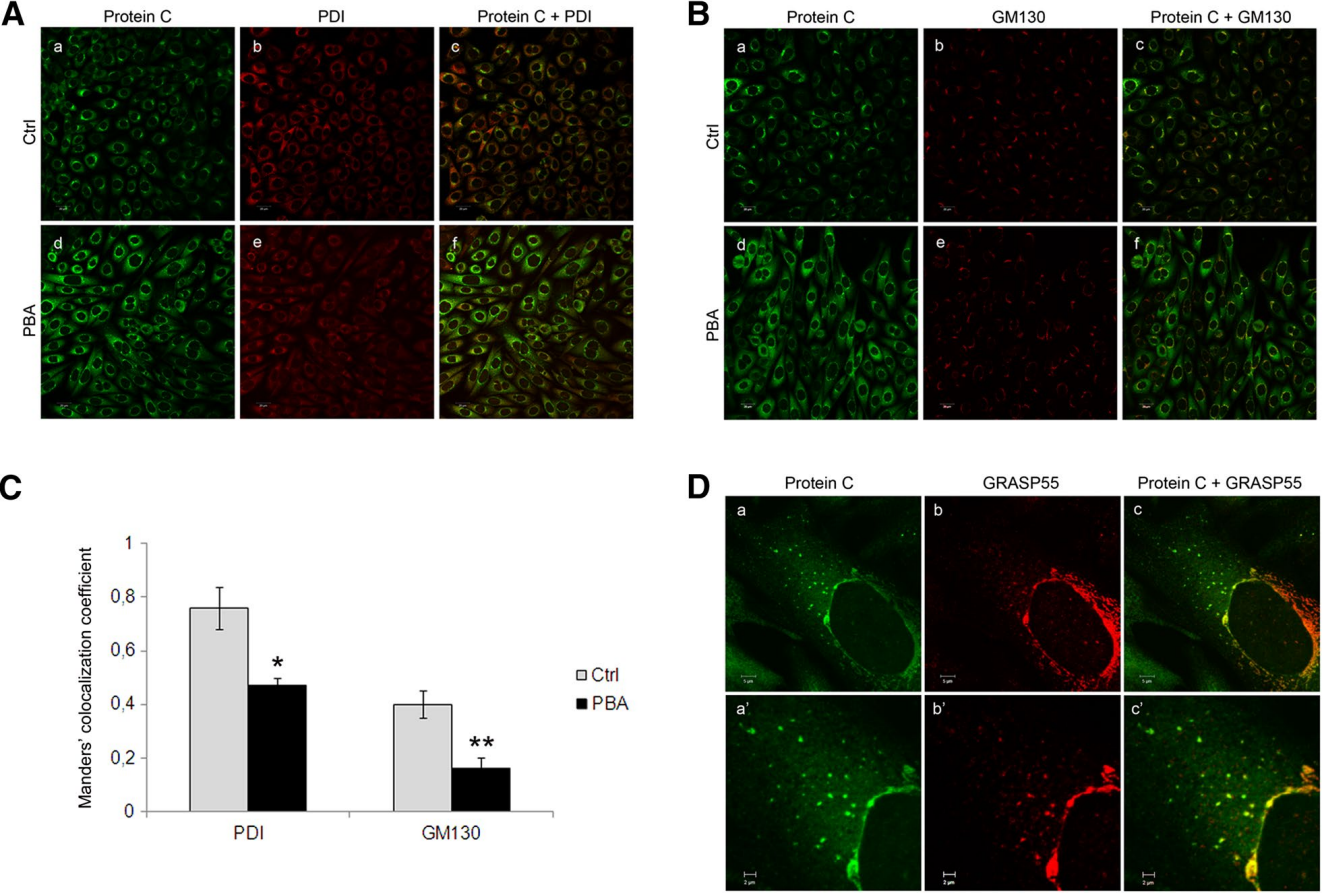
PBA treatment resulted in relocalization of PC_A267T_ from the ER and Golgi to GRASP55-positive vesicles. Confocal images from PC (*green*) and PDI (*red*)-stained (**A**), PC (*green*) and GM130 (*red*)-stained (**B**), or PC (*green*) and GRASP55 (*red*)-stained (**D**) CHO-K1 cells stably transfected with PC-A267T untreated (**A**, **B**, *a*–*c*) or treated with 5 mM PBA for 48 h (**A**, **B**, *d*–*f*, **D**, *a*–*c*). **A** Cells were stained with rabbit polyclonal anti-PC (*a*, *d*), and mouse monoclonal anti-PDI (*b*, *e*), and merged images of *green* and *red* are shown in *c*, *f*. Co-localized *green* and *red* pixels are shown in *yellow* color. **B** Cells were stained with rabbit polyclonal anti-PC (*a*, *d*) and mouse monoclonal anti-GM130 (*b*, *e*), and merged images are shown in *c*, *f*. Three independent experiments were performed. *Bar* 20 µm. **C** The co-localization of PC with PDI or GM130 was calculated based on the merged (*c*, *f*) images in **A** and **B**, by the Manders’ co-localization coefficient. Results are presented statistically as the mean ± SEM of at least three independent experiments. *p < 0.05 Students’ t test comparing PBA-treated cells relative to non-treated (ctrl) cells in the PC/PDI-stained samples. **p < 0.05 Students’ t test comparing PBA-treated cells relative to non-treated (ctrl) cells in PC/GM130-stained samples. **D** Cells were stained with rabbit polyclonal anti-PC (*a*), and mouse monoclonal anti-GRASP55 (*b*). Merged images of *a* and b are shown in *c*, with co-localized *green* and *red* pixels shown in *yellow* color. Zoomed images of the vesicles are shown in *a*′–*c*′. Three independent experiments were performed. *Bar* in *a*–*c* 5 µm. *Bar* in *a*′–*c*′ 2 µm

Interestingly, we observed that the cytoplasmic localization of PC_A267T_ was to some extent associated with vesicles (Fig. 2A, B, d). Since it is known that some proteins can be secreted through unconventional pathways, such as lysosomes and exosomes, phospholipid mediated direct translocation, microvesicles, autophagosomes [8], or the recently discovered GRASP55-dependent pathway [9], we aimed to identify the origin of the vesicles that could potentially be involved in the secretion of the PC_A267T_ mutant. We therefore performed a double staining of PBA-treated cells with PC combined with Rab11 (mouse monoclonal anti-Rab11), Rab8a (mouse monoclonal anti-Rab8a,) or GRASP55 (mouse monoclonal anti-Grasp55). While no co-localization was observed for PC with either Rab11 or Rab8a (data not shown), co-localization was detected for PC and GRASP55, both in vesicles and in the perinuclear areas (Fig. 2D). In the PC_wt_, the presence of some cytoplasmic vesicles that showed co-localization of PC and GRASP55 was also observed after PBA treatment, but to a much lesser extent compared to the PC_A267T_ mutant (Fig. 3). These findings suggest that PBA treatment can promote the secretion of the PC_A267T_ mutant through an unconventional GRASP55 dependent pathway. The role of GRASP55 in such unconventional secretion of certain proteins has been outlined. It was reported in a cell and mouse model of the cystic fibrosis transmembrane conductance regulator (CFTR) ∆F508 mutant, which is misfolded and retained in the ER, that this mutated protein can be transported to the cell surface via a GRASP55 dependent unconventional pathway [10]. In studies in Drosophila cells GRASP has been found at ER exit sites and it has been proposed that GRASP can act as a tethering factor for ER-derived carriers before their fusion with the plasma membrane [9]. By knocking down GRASP proteins, it was shown that they play a role in protein glycosylation and sorting of cargo molecules at the trans-Golgi network [11]. As mentioned above, the involvement of GRASP55 in unconventional secretion of proteins has also been reported in mammalian cells and mice models [10].

**Fig. 3:**
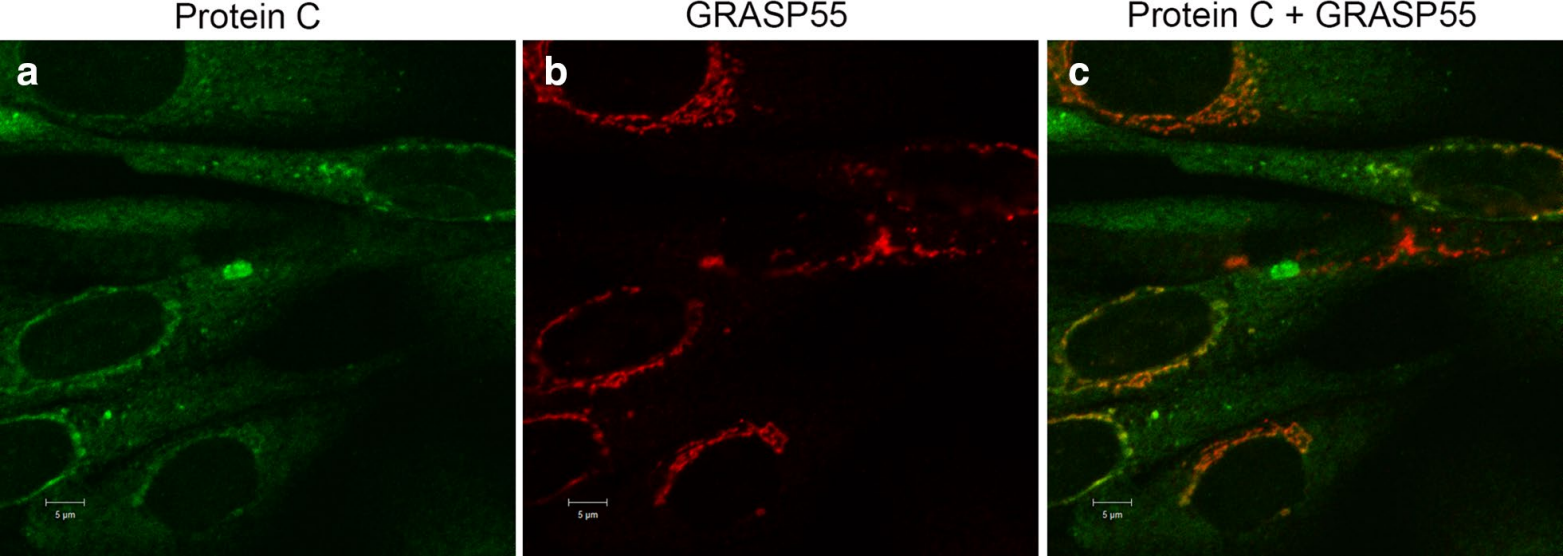
PBA treatment caused some localization of PC_wt_ in GRASP55 positive vesicles. Confocal images from PC (*green*) (**a**) and GRASP55 (*red*) (**b**) stained CHO-K1 cells stably transfected with PC_wt_ and treated with 5 mM PBA for 48 h. Cells were stained with rabbit polyclonal anti-PC (**a**), and mouse monoclonal anti-GRASP55 (**b**). Merged images of **a** and **b** are shown in **c**, with co-localized *green* and *red* pixels shown in *yellow* color. Three independent experiments were performed. *Bar* in **a**–**c** 5 µm

In conclusion, we report here for the first time that PBA improved the secretion of the PC_A267T_ mutant through an unconventional GRASP55 dependent way. Most likely, this is achieved by a process involving stabilization of the protein conformation, which would facilitate the intracellular trafficking of the mutant. Functional studies are required to verify the activity of the secreted PC_A267T_ mutant and also to determine a possible beneficial therapeutic role of PBA in protein C deficiency and other coagulation factor deficiencies in which the genetic defect causes a misfolding of the protein.

## References

[1] Tjeldhorn L, Iversen N, Sandvig K, Bergan J, Sandset PM, Skretting G (2011). Protein C mutation (A267T) results in ER retention and unfolded protein response activation. PLoS One.

[2] Balch WE, Morimoto RI, Dillin A, Kelly JW (2008). Adapting proteostasis for disease intervention. Science.

[3] Tjeldhorn L, Sandset PM, Haugbro K, Skretting G (2010). Hereditary protein C deficiency caused by the Ala267Thr mutation in the protein C gene is associated with symptomatic and asymptomatic venous thrombosis. Thromb Res.

[4] Tjeldhorn L, Iversen N, Sandvig K, Bergan J, Sandset PM, Skretting G (2010). Functional characterization of the protein C A267T mutation: evidence for impaired secretion due to defective intracellular transport. BMC Cell Biol.

[5] Summers RJ, Meeks SL, Healey JF, Brown HC, Parker ET, Kempton CL (2011). Factor VIII A3 domain substitution N1922S results in hemophilia A due to domain-specific misfolding and hyposecretion of functional protein. Blood.

[6] Chaudhuri TK, Paul S (2006). Protein-misfolding diseases and chaperone-based therapeutic approaches. FEBS J.

[7] Burrows JA, Willis LK, Perlmutter DH (2000). Chemical chaperones mediate increased secretion of mutant alpha 1-antitrypsin (alpha 1-AT) Z: a potential pharmacological strategy for prevention of liver injury and emphysema in alpha 1-AT deficiency. Proc Natl Acad Sci USA.

[8] Zemskov EA, Mikhailenko I, Hsia RC, Zaritskaya L, Belkin AM (2011). Unconventional secretion of tissue transglutaminase involves phospholipid-dependent delivery into recycling endosomes. PLoS One.

[9] Giuliani F, Grieve A, Rabouille C (2011). Unconventional secretion: a stress on GRASP. Curr Opin Cell Biol.

[10] Gee HY, Noh SH, Tang BL, Kim KH, Lee MG (2011). Rescue of DeltaF508-CFTR trafficking via a GRASP-dependent unconventional secretion pathway. Cell.

[11] Xiang Y, Zhang X, Nix DB, Katoh T, Aoki K, Tiemeyer M (2013). Regulation of protein glycosylation and sorting by the Golgi matrix proteins GRASP55/65. Nat Commun.

